# Expanding the horizons of medical physics beyond medical radiation science

**DOI:** 10.1002/acm2.14176

**Published:** 2023-10-10

**Authors:** Alhassan Mohammed Baidoo, Stephanie Brako Boateng, Ruth Beulah Awotwe, Samuel Nii Adu Tagoe, Philip Odonkor, Anna Mamoud

**Affiliations:** ^1^ Department of Medical Physics Sweden Ghana Medical Centre, East Legon Hills Accra Ghana Ghana; ^2^ Department of Radiography University of Ghana, East Legon Accra Ghana Ghana; ^3^ Department of Medical Physics National Centre for Radiotherapy and Nuclear Medicine, Korle Bu Teaching Hospital Accra Ghana

Dear Editor,

The vast expanse of medical physics, as a discipline, traverses far beyond the mere application of radiation in healthcare. Yet, there seems to be an inadvertent narrowing of its scope in both academic curricula and public perception. By sequestering the role of medical physics to predominantly radiation‐based applications, we risk overlooking its comprehensive potential in revolutionizing patient care and clinical solutions.

Medical physics involves the application of the concepts and methods of physics to medicine. Its footprint can be observed wherever physics aids in the prevention, diagnosis, and treatment of diseases. This understanding, reinforced by numerous scientists and authors, is also delineated in general literature, from encyclopedias to dictionaries. Consequently, the realm of medical physics extends across every nook and cranny of healthcare facilities. From assessments, such as temperature and pressure‐related vitals, to diagnostic procedures involving medical imaging, and treatments encompassing cardiac defibrillators and artificial heart valves, medical physics plays an instrumental role.

However, the current academic trend is concerning. A majority of graduate programs in medical physics seem to be anchored predominantly on diagnostic imaging, radiation therapy, and nuclear medicine physics. These subjects, while crucial, focus primarily on radiation's clinical, research, and industrial applications. This skewed emphasis has led to a scenario where the term “medical physics” evokes immediate associations with “diagnostic imaging physics, radiotherapy physics, and nuclear medicine physics.” This tunnel vision not only shapes public perception but also manifests itself in the job market. Hospitals tend to list radiologically‐related roles when appointing medical physicists, perpetuating this limited scope.

Through your esteemed journal, we wish to shed light on the need for a broader understanding and application of medical physics, extending beyond the radiation‐centric narrative that currently prevails. We believe that diversifying the roles and responsibilities of medical physicists will significantly contribute to the advancement of healthcare and scientific communities.

Thank you for considering our perspective. We look forward to your action in addressing this urgent matter for the betterment of healthcare and the scientific community at large.

Yours sincerely,

Corresponding author.

## AUTHOR CONTRIBUTIONS

Mr. Alhassan Mohammed Baidoo: Conception of research idea, writing of initial draft, review and approval of finished work. Stephanie Brako Boateng: Conception of research idea and writing of initial draft. Ruth Beulah Awotwe: Conception of research idea and rewriting of initial draft. Samuel Nii Adu Tagoe: Review and correction of written work. Philip Odonkor: Rewriting of initial draft. Anna Mamoud: Rewriting of initial draft.

## CONFLICT OF INTEREST STATEMENT

The authors declare no conflicts of interest.

